# Acknowledgement to Reviewers of *Sports* in 2017

**DOI:** 10.3390/sports6010002

**Published:** 2018-01-11

**Authors:** Sports Editorial Office

**Affiliations:** MDPI AG, St. Alban-Anlage 66, 4052 Basel, Switzerland

Peer review is an essential part in the publication process, ensuring that *Sports* maintains high quality standards for its published papers. In 2017, a total of 94 papers were published in the journal. Thanks to the cooperation of our reviewers, the median time to first decision was 24.5 days and the median time to publication was 56 days. The editors would like to express their sincere gratitude to the following reviewers for their time and dedication in 2017:

Adamczyk, Jakub GrzegorzKirk, BenAerenhouts, DirkKistler, BrandonAndersson, HelenaKiviniemi, Antti MikaelAragon, Alan AlbertKlein, MarkusArdigò, LucaKleiner, Susan M.Aziz, Abdul RashidKnapp, Karen M.Badicu, GeorgianKnechtle, BeatBailey, ChrisKniffin, KevinBarr, MattKnisel, ElkeBarranco Ruiz, YairaKonarski, JanBartlett, MichelleKraus, KorneliusBattino, MaurizioLake, JasonBeckham, George K.Lane, MichaelBergdahl, Celena SchedeLastella, MicheleBermejo, Jose L.Lee, KyuwanBezerra, PedroLee, ElaineBlack, Katherine ElizabethLee, JoohyungBonaiuto, VincenzoLeightley, DanielBotonis, Petros G.Lenoir, MatthieuBottoms, LindsayLisman, PeterBower, RobLiu, ChiangBraakhuis, AndreaLochbaum, MarcBroderick, Tom L.Lopes, Mariana CalábriaBrown, Lee E.Lopez Elvira, Jose LuisBrumitt, JasonLorenzetti, SilvioBryant, ElizabethLow, DanielBurkhart, SarahMalykh, Sergey B.Buschmann, JürgenMamen, AsgeirButton, DuaneMara, JocelynCalmet, MichelMartin, JeffCantarero-Villanueva, IreneMartin, EricCaparrós, ToniMartini, DougCarl, D.L.Mascherini, GabrieleCastillo, DanielMassuça, LuísChan, Roxane RaffinMcArdle, SiobhanChang, Jia-HaoMccoy, StephanieCherry-Allen, KendraMcLean, ScottChhatbar, PratikMcMahon, John J.Chimera, Nicole J.Mehlig, KirstenChiu, Loren Z.F.Melnykowycz, MarkClark, Cain C.T.Millar, Sarah-KateClarke, NeilMiotto, PaulaClarys, PeterMoir, GavinCollado, Pilar SánchezMorouço, PedroComfort, PaulMoya, ManuelConceicao, F.Nachbauer, WernerCools, WouterNavarro, MartinaCourel-Ibanez, JavierNoble, James M.Cremoux, SylvainOlek, RobertCrewther, BlairOliva, PatriziaCuadrado-Peñafiel, VíctorOlson, Michael W.Dabbs, NicoleOxford, SamDalbo, VincentPastor Vicedo, Juan CarlosDanks, MarcellaPereira, AnaDascombe, BenPicone, DeanDatta Banik, SudipPortas, MatthewDeBeliso, MarkPraça, GibsonDeFreitas, Jason M.Rasova, KamilaDevlin, BrookeRattray, BenDillon, Lichar E.Raya-González, JavierDriska, AndyRaynor, AnnetteDrum, ScottRehman, LaureneDuranti, GuglielmoRein, RobertEarnest, Conrad P.Richardson, Sarah PirioEmeljanovas, ArunasRivas, EricEmerson, Dawn M.Robinson, NedEyre, EmmaRogan, SlavkoFairchild, Timothy JohnRomann, MichaelFeigenbaum, Luis A.Rose, Sean C.Felton, L.Ruiz Barquín, RobertoFernandez-Rio, JavierRussell, HayleyFerraz, RicardoSampson, J.A.Fisken, AlisonSandoo, AamerFlora, ColledgeSanz-Rivas, DavidForsell, YvonneSchaffert, NinaFragkos, KonstantinosSchick, EvanFriesen, CarolSchram, BenFry, AndySchubert, Matthew M.Fulford, JonathanSchuermans, JokeGarcia Ramos, AmadorSell, KatieGarten, RyanSenchina, DavidGaume, JohanShadgan, BabakGifford, Janelle A.Sheard, MichaelGómez Ruano, Miguel ÁngelShiraishi, TakehikoGonzalez, AdamSimović, SlobodanGrano, MariaSinclair, PeterGrygorowicz, MonikaSit, CindyGuggenheimer, JoshSlotta-Bachmayr, LeopoldGuglielmino, ClaudiaSmith, StuartGuimarães-Ferreira, LucasSmith, JohnEric W.Gunzler, Douglas DavidSole, Christopher J.Hackett, DanielSpiteri, TaniaHarris, CharlieStanek, AgataHassan, AmrStanforth, Philip R.Hauw, DenisStastny, PetrHeadrick, JonathonStefani, LauraHealy, RobinStorey, AdamHeaney, SusanSuchomel, TimothyHeazlewood, IanTerzis, GerasimosHermanussen, MichaelTorres-Unda, JonHermassi, SouhailTriplett, Travis N.Hillman, Angela R.Trudel, PierreHojman, PernilleTrue, LarissaHosick, PeterUsher, LindsayHowe, SallyUusi-Rasi, KirstiHowie, ErinValovich McLeod, TamaraHritz, NancyVan Den Tillaar, RolandHsieh, Cheng TuVan Hoye, AurélieHsu, Ching-TingVenetsanou, FotiniHwang, AiwenVentura, JessicaIglesias-Soler, EliseoVickers, BradIturricastillo, AitorVoight, MikeJames, Lachlan P.Walker, AnthonyJarrett, KendallWaller, MichaelJones, PaulWang, Jong-ShyanJones, Margaret T.Wang, J.Kam, LynnWang, Shyi-wuKatis, AthanasiosWasik, JacekKavaliauskas, MykolasWeston, KathrynKemper, Han C.G.Wilkinson, Tom J.Kendall, KrissyWillems, MarkKerhervé, HugoYeung, Ella W.Kim, SoJungZając, AdamKing, DougZenic, NatasaKingsley, MichaelZubiaur, MartaKingsley, Derek J.Zuckerman, Scott

